# Custom fabrication and mode-locked operation of a femtosecond fiber laser for multiphoton microscopy

**DOI:** 10.1038/s41598-019-40871-5

**Published:** 2019-03-12

**Authors:** Nima Davoudzadeh, Guillaume Ducourthial, Bryan Q. Spring

**Affiliations:** 1 Translational Biophotonics Cluster, Northeastern University, Boston, Massachusetts, 02115 USA; 2 Department of Physics, Northeastern University, Boston, Massachusetts, 02115 USA; 3 Department of Bioengineering, Northeastern University, Boston, Massachusetts, 02115 USA

## Abstract

Solid-state femtosecond lasers have stimulated the broad adoption of multiphoton microscopy in the modern laboratory. However, these devices remain costly. Fiber lasers offer promise as a means to inexpensively produce ultrashort pulses of light suitable for nonlinear microscopy in compact, robust and portable devices. Although encouraging, the initial methods reported in the biomedical engineering community to construct home-built femtosecond fiber laser systems overlooked fundamental aspects that compromised performance and misrepresented the significant financial and intellectual investments required to build these devices. Here, we present a practical protocol to fabricate an all-normal-dispersion ytterbium (Yb)-doped femtosecond fiber laser oscillator using commercially-available parts (plus standard optical components and extra-cavity accessories) as well as basic fiber splicing and laser pulse characterization equipment. We also provide a synthesis of established protocols in the laser physics community, but often overlooked in other fields, to verify true versus seemingly (partial or noise-like) mode-locked performance. The approaches described here make custom fabrication of femtosecond fiber lasers more accessible to a wide range of investigators and better represent the investments required for the proper laser design, fabrication and operation.

## Introduction

Commercial solid-state femtosecond (fs) lasers are central to the development of nonlinear microscopy as well as its applications to biology and medicine. For instance, these lasers facilitate intravital multiphoton microscopy in neuroscience and in animal models of disease^1,2^. The contemporary commercial solid-state femtosecond laser features exceptional flexibility and ease-of-use with automated alignment and software-controlled tuning over a wide range of wavelengths—especially when paired with an optical parametric oscillator (OPO)^3^. The InSight X3 (Newport Spectra-Physics; <120 fs pulse duration, 80 MHz pulse repetition) and the Chameleon Discovery (Coherent; <120 fs, 80 MHz) are commercial systems that generate 1 W or more of average power over a wavelength range of 680–1300 nm (sources: InSight X3 and Chameleon Discovery data sheets available online). However, the significant cost of a commercial solid-state oscillator (~$100,000) or an oscillator–OPO system (>$200,000) is a major drawback. There are reports of home-built Ti:sapphire oscillators suggesting that custom fabrication can significantly reduce the cost of these devices (Supplementary Note 1). Solid-state ultrafast lasers generally require water cooling and are too bulky and vibration-sensitive to be easily integrated into mobile cart systems ideal for clinical applications (with the exception of solid-state fs lasers presently used in ophthalmologic surgery, Supplementary Note 2).

Fiber lasers are emerging as a potentially reduced cost, compact and high-performance technology to generate short and ultrashort pulses of light. In contrast to solid-state lasers, fiber lasers do not require a chiller or water cooling—air cooling is sufficient due to extended surface area of the waveguide media^4^. In addition, the complex alignment of many of the optical elements traditionally required for the laser cavity is replaced with a simple fusion of fiber components^4^. In fact, all-fiber lasers have been successfully implemented to achieve low-maintenance operation^5–7^ and all-polarization-maintaining-fiber lasers are particularly robust with immunity to environmental factors including vibrations, temperature and humidity^4,8–10^. The potential clinical translation of fiber lasers is exemplified by a mobile cart stimulated Raman scattering microscopy system—featuring a picosecond (ps) fiber laser^11^—for nonlinear, label-free histopathologic imaging of surgical specimens in the operating room^12^. Ultrashort pulse fiber laser technology has matured significantly in the past few years with several commercial products available that are suitable for multiphoton microscopy. The cost of these commercial fs fiber laser systems is typically on the order of $50,000, which is significantly less expensive than commercial solid-state lasers. Outstanding examples of commercial fiber laser technologies include several products offered by KMLabs (<150 fs, 10 MHz and >4.5 W at 1035 nm), Menlo Systems (<150 fs, 100 MHz and >0.3 W at 780, 1030, 1040 and 1560 nm) and Calmar Laser (<120 fs, 80 MHz and >0.5 W at 780, 920 and 1550 nm), where each individual fiber laser generates a single wavelength (sources available online: KMLabs Y-Fi series data sheet; Menlo Systems C-Fiber 780, ELMO, Orange, and YLMO data sheets; and, Calmar Laser Carmel X-series white paper). Presently, these fiber-based systems lack the tunability of solid-state lasers but are more economical for applications that require only a specific wavelength.

Laser physics and engineering have traditionally been inaccessible to the larger community, and it has long been much more efficient to buy a commercial laser system than to invest in the cost, time and personnel to complete a custom build. The potential low cost, simplicity and unique capabilities of fs fiber lasers may ultimately prove attractive for custom design and construction to tailor the performance specifications to the needs of individual laboratories, medical devices and innovative new nonlinear imaging applications^4^. Since the first reports of elegant and high-performance unamplified (<150 fs, 10–100 MHz and >1 W)^13^ as well as amplified (180 fs, 4 MHz, 2 W)^14^ fs fiber laser designs for nonlinear microscopy emerged in 2009–2016^13^, there have been only a few successful attempts at creating custom, high-performance short^11^ and ultra-short pulse fiber lasers^15–18^ and wide spread adoption has yet to catch on. An open question is whether or not custom-fabrication of ultrafast fiber lasers is generally practical by biomedical researchers. The first such report in the biomedical optics community by Perillo *et al*. offers promise with the demonstration of a home-built fs fiber laser that was applied for multiphoton deep brain microscopy in a mouse model^17^. However, the published pulse spectrum and duration data from this first report^17^ indicate that the laser was not operating correctly, as has appeared frequently in the custom fiber laser literature. The performance of these lasers for multiphoton microscopy can be significantly improved with further consideration of fundamental, albeit non-trivial, aspects of the pulse shaping and mode-locking. A key challenge is that partially mode-locked, noise-like pulse generation can be mistaken for fully mode-locked performance in ensemble, time-averaged measurements^19,20^. Just as for solid-state lasers, optimal performance occurs when full mode-locking is achieved—the generation of uniform fs pulse duration and amplitude based on a fixed phase relationship among the cavity modes—to maximize signal-to-background, image acquisition speed and tissue depth during nonlinear imaging applications.

Here, we introduce a cost-efficient and practical approach to custom fabricate a mode-locked fs fiber laser using only readily available components and basic fiber splicing techniques with the motivation of making fs fiber laser technology more accessible to the biomedical research community. We also report laser and pulse characterization methods that minimize cost and complexity but include sufficient diagnostic information to discern partially versus fully mode-locked operation. The resulting fiber laser in this work stably generates mode-locked pulses compressible to 70 fs centered at 1060–1070 nm with a repetition rate of 31 MHz that is compatible with several fluorophores and fluorescent protein sensors used in the life sciences (Supplementary Note 3). A pulse repetition rate of 70 MHz with stable mode-locking was also achieved simply by shortening the fiber ring oscillator circuit length. An average power up to 1 W is emitted directly from the laser cavity without the need for additional amplification. These specifications match those of the commercial solid-state and fiber lasers mentioned above, albeit at a single wavelength.

Note that custom-built fiber lasers may not be suitable for use by scientists and engineers unfamiliar with laser operation and safety. Clearly, commercial systems with an enclosure, turn-key operation and automated alignment provide significant ease-of-use and industrial grade safety features; therefore, commercial femtosecond solid-state and fiber laser systems remain the best choice for many laboratories and core facilities. Furthermore, we describe significant financial and intellectual investments that are required to build a custom fiber laser so that investigators can weigh realistic trade-offs in pursuing custom laser fabrication versus purchasing a commercial system.

## Results

### Low-cost custom femtosecond fiber laser design

**Figure 1:**
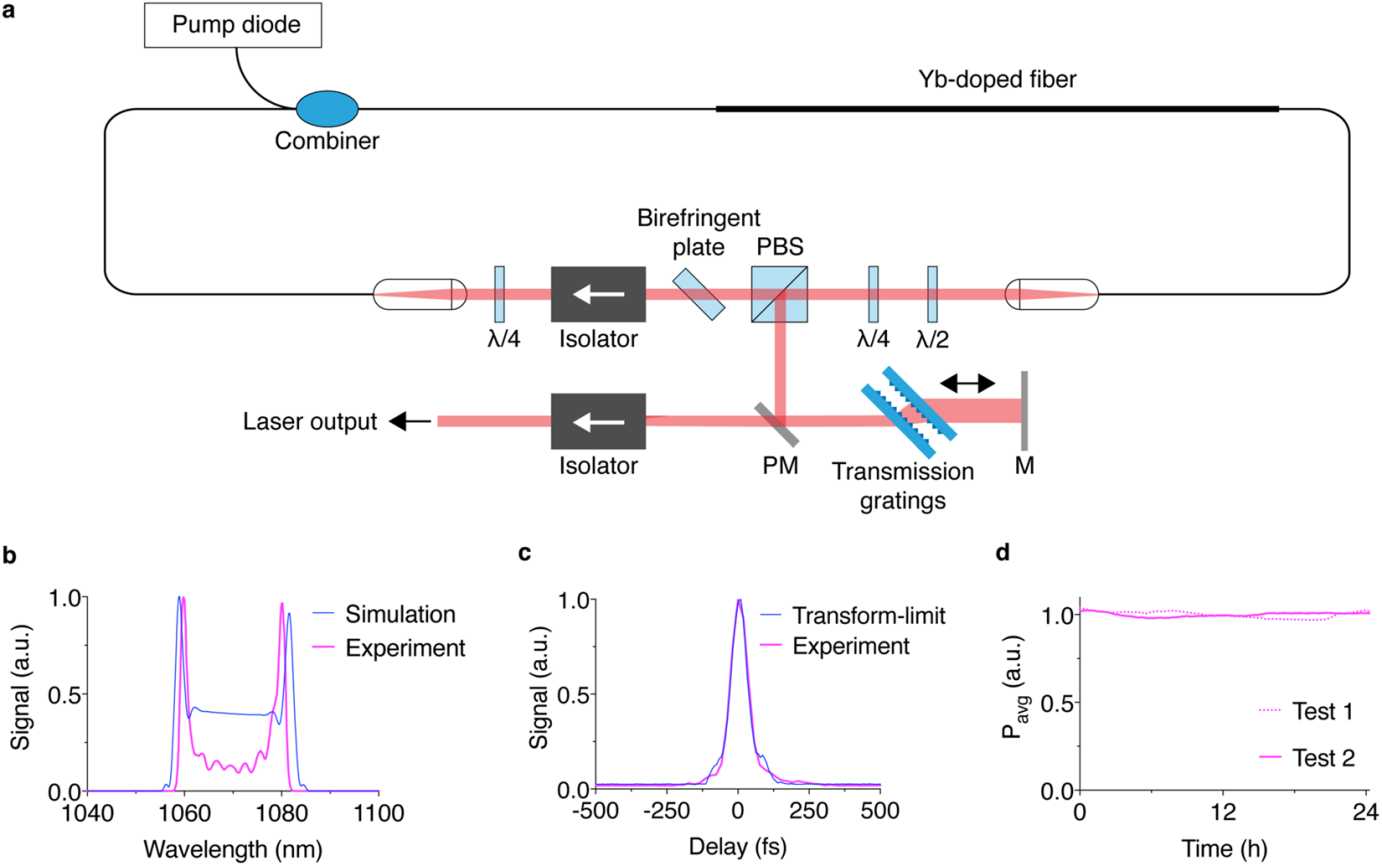
Dissipative-soliton (ANDi) custom fs fiber laser design and basic pulse characteristics. (**a**) Schematic of the laser oscillator and extra-cavity optics for pulse compression and oscillator isolation. λ/2, half-wave plate; λ/4, quarter-wave plate; PBS, polarizing beam splitter; PM, pickoff mirror; M, mirror. (**b**) Simulated (*Methods*) and measured laser pulse spectra. (**c**) Intensity autocorrelation of a 72-fs laser pulse (full-width half-maximum) after dechirping compared to the transform-limited pulse duration based on the empirical pulse spectral bandwidth (25 nm, full-width half-maximum) in **b** (*Methods*). (**d**) Two representative tests of the laser average power stability of the dechirped pulses over a 24 h period.

The custom fs fiber laser (Fig. 1a and Supplementary Fig. 1) was built entirely using readily-available, commercial components (Supplementary Table 1) inspired by all-normal-dispersion (ANDi) dissipative soliton fs fiber laser technology invented by Wise and colleagues^21,22^ that has been commercialized by KMLabs into a rugged, compact and portable system. The ANDi fiber ring oscillator itself provides performance characteristics ideal for multiphoton microscopy without the need for post-cavity amplification when cladding-pumping is utilized with a double-clad gain fiber (as opposed to core pumping)^13^. The simplicity of the ANDi device facilitates its complete construction using off-the-shelf components. ANDi fiber lasers therefore have particular potential for low-cost and custom applications^4,22^.

In addition to the simplicity of the device fabrication, an important distinguishing feature of ANDi is that remarkable intracavity pulse energies (~30 nJ) are supported without distortion^22^. The energy of these dissipative soliton pulses is 1–2 orders of magnitude larger than ordinary or dispersion managed solitons^4,22^. Dissipative solitons accommodate a large nonlinear phase shift (~20π) whereas dispersion managed solitons break apart when the nonlinear phase shift approaches ~π/2, after which the nonlinearity can no longer be balanced by anomalous dispersion^14^. The dissipative solitons accumulate a high chirp (~0.05–0.2 ps^2^) each oscillator round trip due to linear group velocity dispersion and nonlinear phase shift, and fs pulses are achieved by dechirping the pulses to the transform-limited duration outside of the cavity using a pair of transmission gratings.

As is commonly known, mode-locked lasers incorporate an optical element called a saturable absorber that promotes pulsed operation over continuous-wave lasing in the cavity (constant output). In fiber lasers, the high optical intensity inside the fiber can give rise to pulse distortions that significantly reduce their utility. These distortions can be managed through proper design such as the ANDi dissipative soliton mode-locking. The pulse shaping physics of ANDi dissipative solitons elegantly utilizes spectral widening due to concomitant normal dispersion and nonlinear effects within the fiber itself combined with spectral and temporal filtering^22^ (Supplementary Note 4). The major principle of ANDi pulse shaping is that a spectral filter sharpens the highly chirped pulse at the completion of each oscillator round trip by absorbing the blue and red wings of the chirped pulse spectrum—equivalent to cutting the leading and trailing edges of the pulse in time^22^. That is, the free space spectral filter composed of a birefringent plate and a polarization-sensitive isolator (Fig. 1a) dominates the pulse shaping (both spectral and time-domain self-amplitude modulation are controlled simultaneously due to the chirp). Nonlinear polarization evolution in fiber and the free space polarization controller (the waveplates combined with a polarizing beam splitter, Fig. 1a) act as an artificial saturable absorber for self-starting from stochastic fluctuations and also contributes to the self-amplitude modulation. The artificial saturable absorber replaces and outperforms the standard use of a semiconductor saturable absorber mirror, which has a reduced amplitude modulation depth and a low damage threshold that restricts the intracavity pulse energy.

### Low-cost laser fabrication using basic splicing techniques

The Yb-doped gain fiber (single-mode double-clad), the pump and signal combiner output fiber (passive single-mode double-clad) and the single-mode fiber coupled collimators of the ring oscillator were fused using basic fiber splicing equipment (Supplementary Table 2). The mode field diameter of each of the fiber components (Supplementary Table 1) was matched closely in order to minimize the complexity of achieving low loss splices. A plasma fusion splicer with a profile alignment system (programmable core- or cladding-alignment) is required to perform non-homogenous splicing of these dissimilar fiber components within the oscillator, including combinations of single-mode and double-clad fiber. Notably, we applied a simple trick (*Methods*) to achieve reproducible and reasonably flat (0 ± 0.2°) cleave angles using an inexpensive fiber cleaver (Supplementary Table 2) that are otherwise not possible without more sophisticated equipment. The level cleave angle is important for minimizing reflections at the fiber splice joints that can seed amplified spontaneous emission leading to fiber damage. Here, we selected a 6 μm-core-diameter Yb-doped double-clad fiber such that the gain fiber contributes to the overall accumulation of nonlinear phase shift within the cavity, which helps to stabilize mode-locking. In contrast, many prior designs have utilized larger core diameters with significantly reduced nonlinearity^13,17^. The choice to use a 5 μm-core-diameter double-clad fiber for the pump and signal combiner was based on its availability off-the-shelf to reduce cost. This is a sensible trade-off since the mode field diameter mismatch has minimal impact. The combiner output fiber guides the multimode pump light into the double-clad gain fiber, which has a larger cladding numerical aperture to minimize loss, and the combiner input fiber contributes ~6.3% (<0.3 dB) single mode coupling loss to the oscillator circuit at the splice joint between the single-mode fiber (from the collimator) and the single-mode, passive-double-clad signal input fiber of the combiner. The two other splices within the oscillator are low-loss (estimated to be <0.07 dB loss per splice, or ~98.5% transmission; Supplementary Fig. 2) for a total oscillator splice transmission of ~91% (0.4 dB loss). Therefore, tapering is not required for any of the splices.

The total length of the fiber ring is 6.5 m, plus approximately an additional 0.4 m path length through the free space optical components (Supplementary Fig. 3), for a pulse repetition rate of 31 MHz. This design includes sufficient nonlinear phase accumulation to comfortably facilitate stable mode-locking. Remarkably, we were able to achieve a pulse repetition rate of 70 MHz without any modifications to the laser oscillator other than shortening the length of the combiner and collimator fiber prior to the gain fiber along the pulse trajectory (Supplementary Fig. 4). Other repetition rates are possible in general by balancing group velocity dispersion with the spectral filter bandwidth and nonlinear phase shift^21^.

### Basic fiber laser characterization

Once constructed, we first measured the laser spectrum emitted from the cavity using an optical spectrum analyzer while adjusting the waveplates to find mode-locked operation at pump powers sufficient to induce nonlinear polarization evolution (*Methods*). In the present design the oscillator output is just before the spectral filter to allow diagnostics on the amplified, highly chirped pulse. This is advantageous because the fine structure of the amplified, chirped pulse spectrum (the “cat ear” or “Batman” shape) are signatures of ANDi operation^22^ and provide an indicator to guide the search for mode-locked operation (Fig. 1b). The steep sides of the spectrum are due to nonlinear self-phase modulation as predicted by analytical and numerical simulations of the laser operation^22^, and this characteristic spectrum is required for proper laser operation. As an aside, it is of course possible to place the output, or to add a second output, after the spectral filter to obtain a smoother spectrum and a cleaner temporal pulse by sacrificing pulse energy^4,22^. Next, we verified the generation of ultrashort pulses using an intensity autocorrelator to measure the pulse duration before and after dechirping. These measurements confirm that chirped ps pulses (~2 ps) are output directly from the cavity and that they are compressible to ultrashort durations. We routinely achieved pulse durations of ~70 fs (full-width half-maximum of the intensity autocorrelation data without fitting or assuming a pulse shape, Fig. 1c) using a simple transmission grating pair for group velocity (second-order) dispersion compensation and pulse compression. The dechirped pulse duration approaches estimates of the transform-limited compression (Fig. 1c; 69 fs for the measured 25 nm laser spectral bandwidth in Fig. 1b; *Methods*).

The stability of low-cost, custom-built systems has rarely been characterized quantitatively such that an open question is whether or not these home-built systems are suitable for research. Therefore, as a further basic diagnostic we measured the average power and spectral stability of the dechirped pulses over a 24 h period using the optical spectrum analyzer. Here, the entire laser was constructed on a breadboard (Supplementary Fig. 1) resting on an active floating optical table to damp mechanical vibrations. Under these standard laboratory conditions, the laser output stability is suitable for routine imaging experiments (< ±3.5% power drift over the 24 h period, Fig. 1d). The power stability is concomitant with a stable spectral profile over the same period (Supplementary Fig. 5). We find that the laser remains stable and self-starting for a period of a week or more even when powered down between experiments. The free space components undergo mechanical drift and the mode-lock is lost over several weeks but can be re-gained in a matter of minutes by simple readjustments.

### Application to multiphoton microscopy

**Figure 2:**
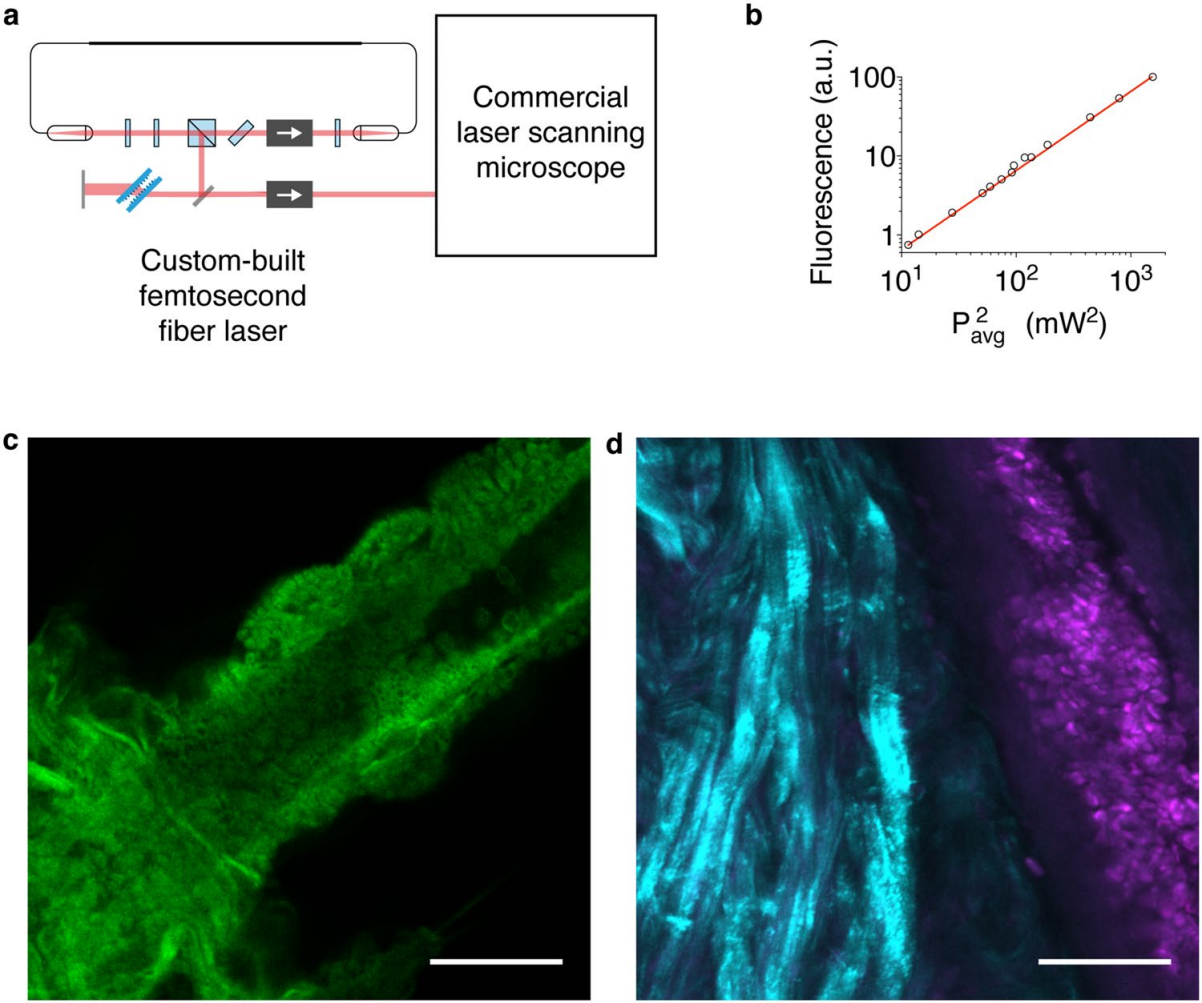
Multiphoton excitation of fluorescence and laser scanning microscopy using the custom fs fiber laser. (**a**) Schematic of the custom-built fiber laser output directed into the beam combiner and laser scanner of a commercial confocal microscope to acquire images. (**b**) Multiphoton excited fluorescence plotted versus the square of the average excitation power. The trendline is a quadratic fit indicating a two-photon excitation process (linear for the double logarithmic plot against squared power). (**c**) Multiphoton excited autofluorescence from an unstained, fixed brine shrimp sample. (**d**) Second harmonic generation from collagen fibrils (cyan) and multiphoton excited fluorescence from fluorescently-stained cells (magenta) in a freshly excised chicken tissue specimen stained with rhodamine B. Scale bars, 50 μm.

Measurement of a nonlinear process is the ultimate test to verify sufficient pulse peak-power for practical use. Therefore, after attaining stable pulse generation as described above, we next coupled the dechirped output of the custom fiber laser into a commercial laser scanning microscope to perform multiphoton excitation imaging (Fig. 2a). Here, de-scanned detectors were used to collect images without modifying the microscope, and more efficient signal collection is of course possible using non-descanned detectors. The detector pinholes were fully opened in order to maximize signal collection. First, images of a fluorescent dye solution (Alexa Fluor 568) were captured while varying the laser power with a set of neutral density filters. The resulting fluorescence signal (*F*) indicates the expected quadratic dependence on the average laser power (*P*_*avg*_) for a two-photon excitation process (Fig. 2b). This proportionality may be expressed as $F\propto \,\delta \times {P}_{avg}^{2}/(f\times \tau )$ (Eq. 1) to emphasize the pulsed laser-dependent parameters, where the inverse product of pulse repetition rate (*f*) and duration (*τ*) is key for enabling efficient two-photon excitation at average powers safe for use in living subjects despite the relatively weak nonlinear absorption cross-section (*δ*) of fluorophores compared to linear excitation^23^. This factor is the laser duty cycle and typically reaches 10^5^ for commercial solid state lasers (*e.g*., 100 fs pulses at a rate of 100 MHz), which corresponds to peak powers of ~10–30 kW at typical average powers safe for deep *in vivo* multiphoton imaging (*e.g*., up to ~100–300 mW can be delivered safely to the surface of a mouse brain with the focus at depth)^17,24^. This corresponds to a laser source that provides at least 1 W of average power (100 kW peak power) to account for lossy microscope optics.

**Figure 3:**
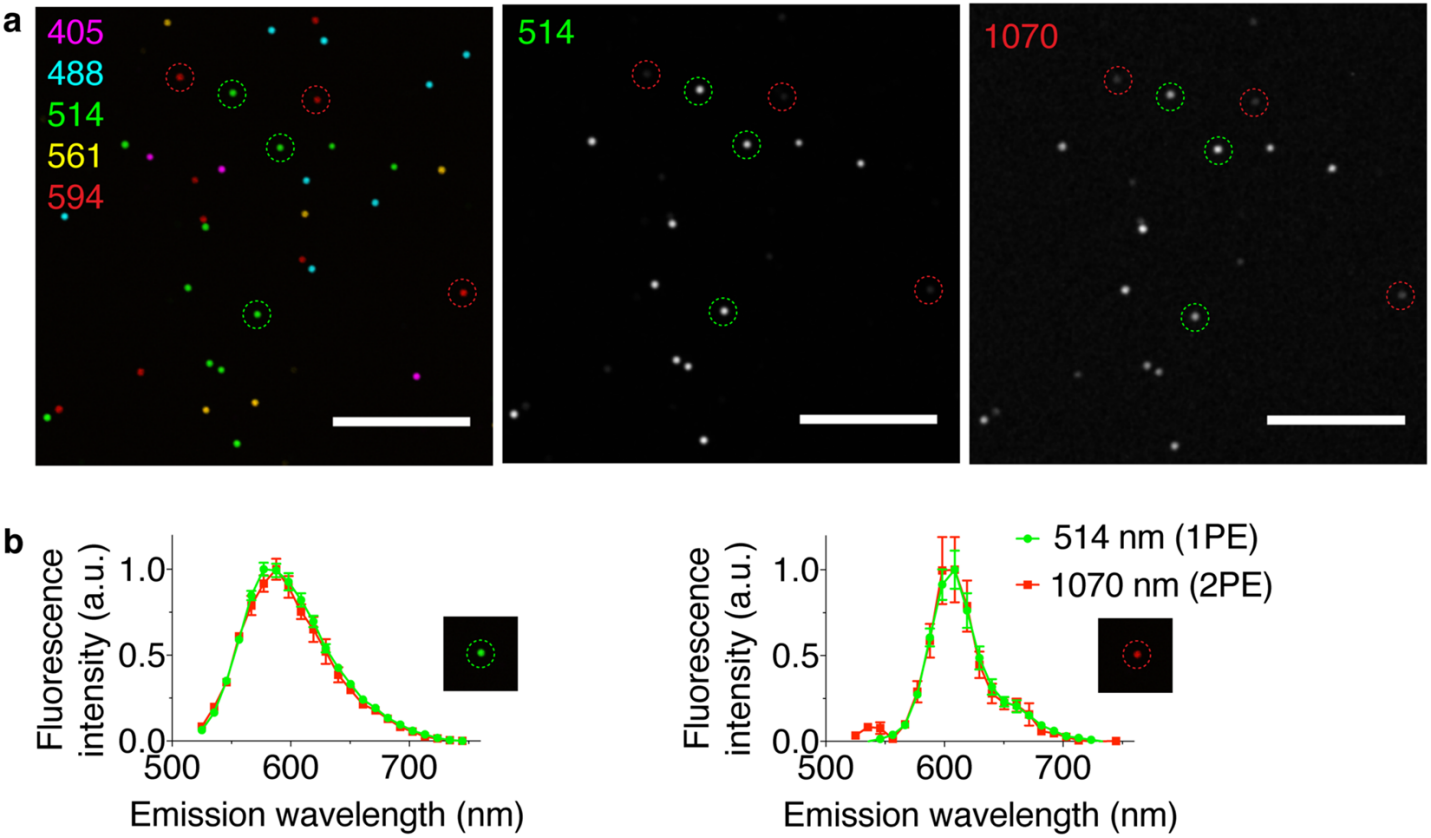
Multiphoton excitation using the custom fs fiber laser versus single-photon excitation with a commercial laser diode. (**a**) Linear excitation of 5 fluorescent microspheres (3-μm-diameter, false colored according to the linear excitation wavelength) using a set of 5 laser diodes in sequence (405, 488, 514, 561, and 594 nm) to collect a 5-channel image (*left*). A maximum intensity projection of a hyperspectral image cube acquired using the 514 nm diode to linearly excite a subset of 2 fluorescent microsphere populations (*middle*). The maximum intensity projection of a hyperspectral image cube acquired using the custom fs fiber laser for multiphoton excitation centered at 1070 nm (*right*) generates fluorescence from the same subset as linear 514 nm excitation. Scale bars, 50 μm. (**b**) Normalized fluorescence emission spectra from analysis of the linear 514 nm and nonlinear 1070 nm excited green (*left*) and red (*right*) fluorescent bead populations. Results are mean ± s.e.m (*n* = 3 fluorescent microspheres per data point; *i.e*., the 3 green and the 3 red fluorescent microspheres outlined with dashed circles in **a**). The short peak at 535 nm in the two-photon excitation spectra of the red bead results from a weak harmonic of the 1070 nm excitation light.

The custom fiber laser and laser scanning microscope successfully collected images of multiphoton excited tissue autofluorescence (Fig. 2c) as well as second harmonic generation from collagen fibrils and multiphoton excited fluorescence from fluorescently-stained cells (Fig. 2d). Hyperspectral images of multicolor fluorescent beads were also collected using the custom fiber laser, and the fluorescence emission spectra of two of the bead colors excited with the custom-built laser were compared to linear excitation with a commercial diode laser (OBIS 514 nm, Coherent). The fluorescence emission spectra resulting from linear and nonlinear excitation are identical (Fig. 3), as expected for a two-photon excitation process using the custom fiber laser. Collectively, these results indicate that the custom fiber laser generates pulses with peak power capable of generating two-photon excited fluorescence and second harmonic generation.

### Characterization of mode-locked versus seemingly mode-locked performance

A critical issue is that the above diagnostics are insufficient to verify mode-locking and optimal laser performance. The challenge is that it is possible to obtain seemingly mode-locked pulse trains and even multiphoton images in what is often described as noise-like or partially mode-locked operation^19,20,25^. Both mode-locked and partially-mode locked operation produce a regular pulse train with a repetition rate determined by the cavity length. A partially mode-locked oscillator will output a seemingly regular pulse train with fluctuations in the pulse spectra and duration due to pulse-to-pulse phase incoherence, whereas mode-locked operation generates pulses with nearly completely locked phase coherence.

**Figure 4:**
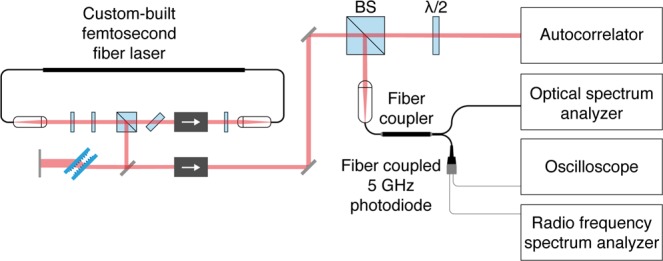
Ultrafast pulse diagnostics setup to discern various modes of fs fiber laser operation. Schematic of the custom-built setup using common equipment to measure the pulse duration (autocorrelator), the pulse spectrum (optical spectrum analyzer), the pulse train in time (oscilloscope), and the pulse train frequency power spectrum (radio frequency spectrum analyzer).

Single-shot measurements and direct real-time pulse characterization would be ideal for pulse diagnostics but are intractable due to the fast repetition rates (MHz) and ultrashort pulse durations (fs). Consecutive pulse interference and other methods have been developed to reveal the features of partially mode-locked pulses^19,20,25^, however, these methods add complexity and cost to design and to custom-build the diagnostic tools. Here, we use commercially available equipment (Supplementary Table 2) to discern mode-locked (ML), partially mode-locked (PML) and non-mode-locked (NML) operation of the custom fiber laser. A simple setup was built to run the diagnostic measurements in parallel (Fig. 4), including: the time-averaged pulse spectrum (~125 pm resolution); the time-domain pulse train (~200 ps resolution); the radio frequency (RF) spectrum (or power spectrum) of the pulse train (with a resolution bandwidth of 10 Hz and a span range of 100 kHz), which characterizes fluctuations in pulse repetition rate, intensity and duration; and, the pulse duration via intensity autocorrelation in which two copies of the incoming pulse train are superimposed and scanned through a variable temporal delay to create a nonlinear response when they overlap in time (<5 fs resolution).

**Figure 5:**
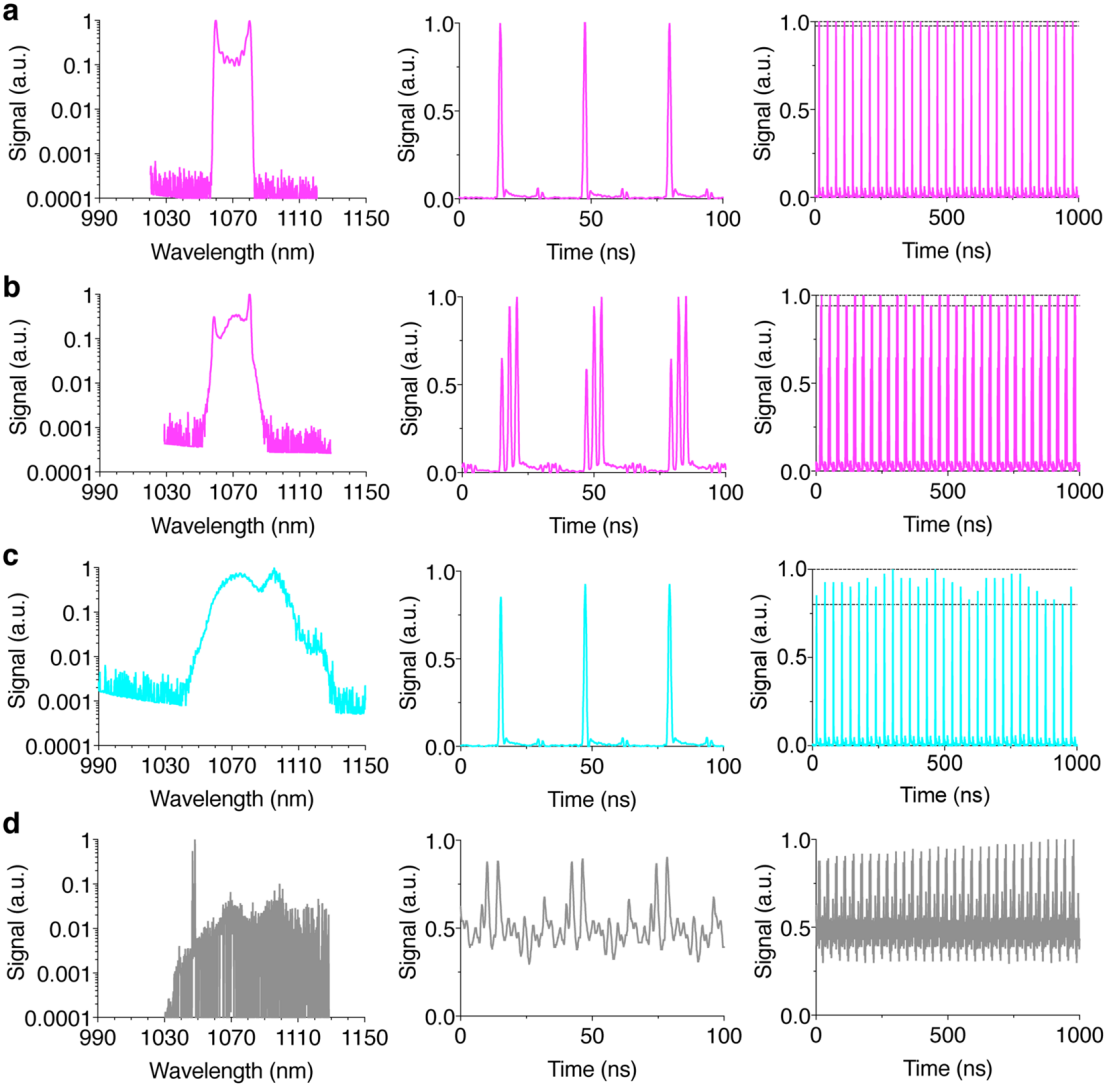
Pulse spectra and temporal pulse trains illustrate several exemplary modes of fiber laser operation. (**a**) Pulse spectrum (*left*) and pulse trains (*middle* and *right*) for single-pulse ML operation. The dotted lines (*right*) indicate pulse-to-pulse amplitude fluctuations of ±1.25%. (**b**) Multi-pulse ML operation with pulse-to-pulse amplitude fluctuations of ±3% (dotted lines, *right*). (**c**) PML operation with pulse-to-pulse amplitude fluctuations of ±10% (dotted lines, *right*). (**d**) NML operation demonstrated using unstable Q-switching as an example with >100% pulse fluctuations over longer time scales (not shown).

**Figure 6:**
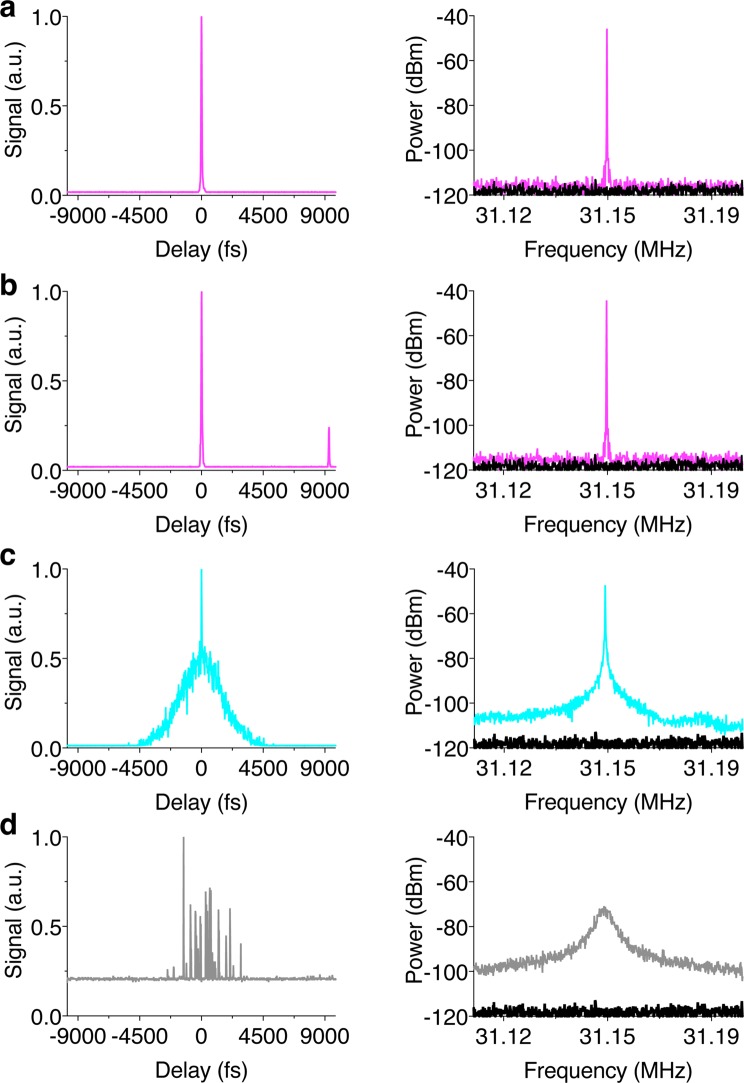
Pulse duration and power spectra discern several modes of custom fiber laser operation. (**a**) Pulse intensity autocorrelation (*left*) and radio frequency power spectrum (*right*) for single-pulse ML operation. (**b**) Multi-pulse ML operation. (**c**) PML operation. (**d**) NML operation demonstrated using unstable Q-switching as an example. The noise floor is shown in black for each of the power spectra.

These diagnostic measurements illustrate key signatures for distinguishing the various modes of operation. First, single-pulse ML operation shows the characteristic “cat ear” spectral structure of ANDi pulse shaping and a regular pulse train with small amplitude fluctuations (Fig. 5a). At higher pump powers (>3.6 W for the present laser design), stable multi-pulse ML operation is evident in the measured pulse train due to pulse energy quantization, saturation and bifurcation into multiple pulses each with reduced energy^26,27^ (Supplementary Note 5). In the example here, the characteristic ANDi “cat ear” spectrum is apparent for multi-pulsing ML with increased but still minimal pulse amplitude fluctuations (Fig. 5b). Note that the pulse bifurcation is not necessarily detrimental to imaging as long as sufficient peak power for multiphoton excitation is delivered to the focus and the mode-lock is stable. In fact, high degrees of pulse splitting (~100×) are desirable at higher laser powers to enhance imaging speed by reducing prohibitive increases in nonlinear photodamage as the laser power is increased^28^. The intensity autocorrelation traces for both single- and multi-pulse ML indicate a clean time duration profile suggesting proper laser function (Fig. 6a,b), whereas pedestals are often visible in the pulse autocorrelation trace in the absence of mode-locking. A second pulse is visible at the edge of the autocorrelation trace bandwidth for multi-pulsing due to the close proximity of a second pulse (Fig. 6b). The radio frequency power spectra confirm the stability of the output pulse train for both single- and multi-pulse ML with the absence of sidebands and harmonic frequencies to at least 70 dB below the pulse repetition fundamental frequency (Fig. 6a,b), limited by the dynamic range of the RF spectrum analyzer.

In contrast, a signature of PML is that the ensemble laser spectrum is relatively smooth (Fig. 5c) and lacks the steep sides (“cat ear” shape) of ML operation. The smooth spectral profile of PML operation results from time-averaging of the fluctuations among highly structured individual pulse spectra^19,20^. The PML pulse train indicates a regular pulse train with significant amplitude fluctuations (Fig. 5c). The time-domain pulse autocorrelation of PML operation contains a fine fs peak feature that rests on a broad pedestal of ps duration (Fig. 6c). The fs peak arises from partial coherence within the substructure of the PML pulses^19,20^, and this feature appears similar to ML operation. However, the broad pedestal is a distinct signature of PML. Similarly, the PML power spectrum contains a peak at the cavity repetition rate sitting on a pedestal corresponding to fluctuations in pulse amplitude and duration (Fig. 6c).

Finally, NML operation is a generic term and here we use unstable Q-switching as an example with many continuous wave (CW) modes that are not phase locked. Other modes of NML operation are possible including those that involve amplified spontaneous emission. Here, unstable Q-switching operation generates a range of pulse durations (from femto- to picosecond) due to nearly completely random modes. NML operation unambiguously contrasts strongly with ML operation for each of the diagnostic tests (Figs 5d and 6d). Many CW modes are visible in the broad laser spectrum for NML Q-switched operation (Fig. 5d). The NML Q-switched pulse train (Fig. 5d) as well as its pulse autocorrelation and power spectrum (Fig. 6d) show large fluctuations in pulse amplitude, duration and repetition with highly populated sidebands surrounding the fundamental pulse repetition rate.

### Mode-locked pulse generation provides optimal imaging performance

**Figure 7:**
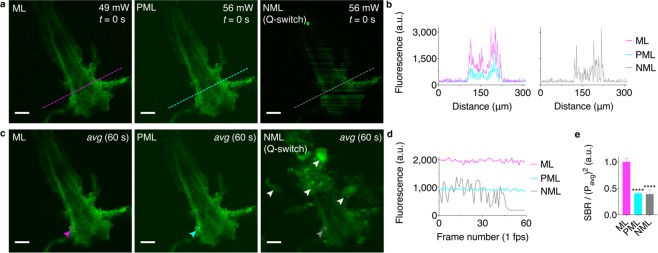
Mode-locking increases multiphoton excited fluorescence signal compared to partial mode-locking and non-mode-locked operation. (**a**) Snapshots from the start of a 60 s time series of images (*t* = 0 s) acquired at 1 frame (512 × 512 pixels) per second for ML, PML and NML (unstable Q-switching) fiber laser operation (Supplementary Videos 1–3). The sample is an unstained, autofluorescent brine shrimp specimen. (**b**) Line profiles corresponding to the dashed lines in** a** indicate varying signal and noise levels for each mode of operation. Streaking and noise are evident from stochastic pulsing during NML (Q-switching) imaging in a and the line profile in **b**. (**c**) Mean intensity projections over the full image time series. White arrows in **c** (NML) indicate regions with significant photodamage. (**d**) Mean fluorescence signal over the image time series corresponding to a group of 10 × 10 pixels demarked by the colored boxes and arrows in **c**. Stochastic pulse amplitude and duration during NML (Q-switching) operation results in significant photodamage and a loss of signal by the end of the time series. (**e**) The signal-to-background ratio normalized by the squared average power for ML, PML and NML (Q-switching) operation. Results are mean ± s.e.m (*n* = 5 regions of 10×10 pixels with tissue autofluorescence signal divided by 5 regions of 10 × 10 pixels with background dark current for each image in in **a**). Asterisks denote significance compared with ML operation (****P < 0.0001, one-way ANOVA with Tukey’s *post hoc* test). The PML and NML images are brightened for visibility with respect to the ML images in (**a**–**c**). Measurement conditions (laser output intensity autocorrelation pulse duration, average power at sample): ML, 112 fs, 49 mW; PML, ~100 fs peak feature with ~2 ps pedestal, 56 mW; NML (Q-switch), ~40–120 fs fluctuations with a continuous wave background, 56 mW. Scale bars, 50 μm.

We collected image time series (Supplementary Videos 1–3) using each of the exemplary modes of laser operation described above (Fig. 7). ML operation provides the brightest images as expected (Fig. 7a–e). Remarkably, PML operation does produce fluorescence signal (Fig. 7a–c) but ~2.5× less efficiently than ML operation in this example (Fig. 7d,e). The sporadic generation of fs pulses is apparently sufficient to produce fluorescence signal because many pulses are delivered to the sample per pixel (~118 pulses/pixel; 3.8 μs/pixel, 31 MHz pulse repetition rate). However, a number of ps pulses are also delivered that lack sufficient peak power for multiphoton excitation, thus, the signal is reduced. That is, the laser average power is divided among fs and sub-fs pulses such that multiphoton excitation is much less efficient for PML operation. Finally, NML operation with Q-switching instability leads to sporadic generation of multiphoton excited fluorescence (Fig. 7a,b) as well as excessive photodamage due to large bursts in pulse peak power during the image time-series (Fig. 7c,d; Supplementary Video 3).

## Discussion

To the best of our knowledge, the present laser design is the lowest cost custom-fabricated, ultrafast fiber laser oscillator ever reported with performance specifications suitable for multiphoton microscopy without the need for post-cavity amplification (Supplementary Table 3). In comparison to the prior report of a custom-built ANDi fiber laser for $13,000 in major components^17^, the part list and total cost reported here include several items required for the laser construction that were previously omitted; *i.e*., an additional isolator outside of the oscillator ring that is essential to attenuate back reflections from the microscope optics that otherwise disturb mode-locking as well as a number of standard optical components (Supplementary Table 1). Excluding the previously omitted components for a direct comparison, the price of the major components for the present design is $9,800—a 25% reduction in expense. In addition, the fiber laser fabrication protocol presented here utilizes simple fiber cleaving and splicing techniques and equipment (Supplementary Table 2) for fusing dissimilar fiber compared to prior reports that utilized more expensive equipment. This is impactful for researchers who will need to build new infrastructure for fiber optic fabrication and who otherwise do not have access to this equipment. The cost of the fiber cleaving and splicing equipment used here was $38,000 (Supplementary Table 2). Note that a valuable resource for custom fs fiber laser fabrication is an outstanding and comprehensive description of a home-built frequency comb fiber laser system for ultrafast spectroscopy by Allison and colleagues using simple splicing techniques^16^. In contrast to the present design of a high-power oscillator to minimize cost and complexity, the frequency comb fiber laser system consists of a low power, single-mode pumped ANDi oscillator (38 mW) with significant effort and additional components to perform extra-cavity fiber amplification (155 fs, 87 MHz and 80 W at 1035 nm)^16^. The methods developed here uniquely make the fabrication process of a high-power oscillator more accessible to researchers new to fiber optics.

Once the fiber laser is assembled, the attainment of stable mode-locking is critical for its practical use and application to multiphoton microscopy. Therefore, the present work also contributes basic concepts and protocols to distinguish the major classes of laser operating modes. The set of pulse diagnostic techniques outlined above are sufficient to optimize mode-locking and to recognize sub-optimal laser performance. Here, we translated tools common in the laboratory of the fiber laser inventor into a simple setup and procedures that minimize cost and that may be more readily utilized by the community ($25,000 in equipment, Supplementary Table 2). Clear signatures of ML versus noisy pulse generation are presented as a guide. Note that the operation modes of the laser shown here are representative of a continuum of possibilities among full mode-locking, partial mode-locking and stochastic operation. Therefore, other results from the pulse diagnostic tests are possible. However, the crux is that it is possible to clearly distinguish partial from full mode-locking—addressing the major pitfall to mistake the regular, seemingly mode-locked pulse train produced by partial mode-locking as full mode-locking.

The ANDi laser design presented here is one of a few established fs fiber laser designs. ANDi features a particularly simple physical design that makes fabrication accessible, and cladding-pumped oscillators can deliver powers sufficient for multiphoton imaging applications without the need for further amplification. Therefore, we encourage others to first build this very same design to familiarize themselves with the entire custom-building process. A limitation of ANDi is that nonlinear polarization evolution is not compatible with polarization-maintaining fiber (the ideal fiber for environment stability). This means, in practice, that ANDi lasers are not intrinsically immune to vibrations and temperature fluctuations without effort to mechanically fix the fiber components and to implement air cooling (as achieved by KMLabs). In contrast, fs fiber laser designs based on the nonlinear optical loop mirror (NOLM) mode locking mechanism have been implemented in polarization-maintaining fiber (with some free space components) as commercialized by Menlo Systems (also known as the figure 9 oscillator)^29^. The NOLM oscillators are low power and achieve performance ideal for imaging using post-cavity amplification in one or two stages.

While the ANDi design presented here represents the peak performance expected from an easily-constructed, low-cost fiber oscillator, it is worth noting that the state-of-the-art in mode-locked fiber oscillators is steadily progressing. In the near future, we anticipate devices with similar ease-of-construction and maintenance with order-of-magnitude increases in pulse energy, several-fold decreased pulse duration and complete environment stability. For instance, the Mamyshev oscillator has recently emerged as a next-generation fs fiber laser technology that is compatible with polarization-maintaining fiber and that achieves extraordinary features, including an order of magnitude increase in peak power compared to ANDi and other fiber laser designs (>1 MW peak power, 35 fs pulses, 17 MHz)^30,31^. Wise and colleagues most recently reported the innovation of a self-seeded Mamyshev oscillator, which is a promising advance towards making this new laser design practical^31^. Knowledge and experience fabricating ANDi lasers will enable adoption of these emerging techniques—the majority of the Mamyshev physical device consists of the same parts used to construct the ANDi laser. All of these fiber laser systems now provide power levels sufficient to drive multi-wavelength systems with synchronized pulses using wavelength-shifting techniques^32–34^, which will pave the way towards the development of novel ultrafast, nonlinear imaging techniques.

Custom-fabrication of ultrafast fiber lasers has emerged as an attractive venture for biomedical researchers to develop low-cost yet high performance devices for multiphoton microscopy and endoscopy. However, the early attempts have demonstrated that much more know-how is needed than just a parts list. Here, the point is not just demonstration of a one-time build but rather that an intellectual investment in understanding the laser physics can lead to practical construction of high-performance devices. Clearly, a significant—but not impractical—up-front financial investment is required for those without access to fiber splicing and pulse diagnostic equipment. We anticipate that broader adoption of custom-fabrication by researchers at the frontiers of novel biomedical applications will bridge new advances in laser physics with more immediate practical use in applied research. This may also accelerate the development and impact of novel medical devices for imaging and photomedicine that leverage the new capabilities of portable ultrafast fiber lasers.

## Methods

### Simulations

The custom fiber laser designs with 31 MHz and 70 MHz pulse repetition rates (Supplementary Figs 3 and 4) were both simulated in MATLAB by adapting scripts from those in the literature^35^ and applied successfully in prior reports^21,22,30,31,36,37^. Briefly, numerical simulations of the laser pulse shaping (including the simulated pulse spectra shown in Fig. 1b and Supplementary Fig. 4b for comparison to experiment), were conducted using the split-step Fourier method^38^ to solve the nonlinear partial differential equation (a generalized nonlinear Schrödinger equation) that models ANDi pulse evolution, linear and nonlinear dispersion as well as dissipative processes and other cavity effects such as the spectral filter, saturable absorber and so forth^22^. The dispersion characteristics of the specific optical fibers utilized in the present design were included in the simulations. The transform-limited pulse duration (Fig. 1c) was computed in MATLAB by calculating the inverse fast Fourier Transform of the measured pulse spectrum (Fig. 1b). That is, the measured spectrum was first interpolated and converted to frequency units (*interp1* function), and the square root of this function produces the frequency-domain electric field, *E*(*ω*). The time-domain, transform-limited pulse intensity, *I*_*TL*_(*t*), then results from the square of the inverse Fourier Transform (*ifft* function) of *E*(*ω*), ${I}_{TL}(t)={|ifft\{E(\omega )\}|}^{2}$ (Eq. 2). Finally, the autocorrelation of the resulting transform-limited pulse was obtained via convolution (*conv* function) for comparison with the measured pulse intensity autocorrelation (Fig. 1c).

### Fiber splicing

In order to reduce costs, we used an inexpensive fiber cleaver (Supplementary Table 2) as opposed to more advanced systems that accommodate non-circular fiber (such as the Vytran LDC401, Thorlabs). The V-groove of the fiber cleaver has a round shape while the double-clad fiber is hexagonal, and this mechanical mismatch leads to instability and a lack of reproducibility in the cleave angle. A simple trick was employed to achieve reproducibly flat cleave angles for double-clad fiber. First, we cleaved the double-clad fiber by hand and then spliced the same fiber to circular 125-μm-cladding single-mode fiber. The single-mode fiber portion is then used to hold the fiber in the V-groove to achieve a flat cleave angle prior to splicing. Fusion splicing was then carried out according to standard protocols, and we found it helpful to first practice each splice with extra fiber to optimize the instrument settings.

### Mode-locking and wavelength tuning

A search for mode-locking is performed efficiently as described by Allison and colleagues^16^ and in a video tutorial (Supplementary Note 6). Briefly, adjustments are made by systematic rotation of the waveplates while monitoring the spectrum (optical spectrum analyzer) and pulse train (oscilloscope) of the amplified, highly chirped pulse as the primary, real-time indicators of mode-locking (followed by further pulse diagnostics). First, the alignment of the collimators and the intermediary optical elements within the oscillator circuit (including the waveplates and isolator) should be tuned to minimize the pump power required for lasing (*i.e*., continuous-wave laser output). After this is achieved, the first quarter-waveplate in the circuit (rightmost, Fig. 1a) need not be adjusted further. The pump power is then increased beyond the threshold required for nonlinear polarization evolution and mode-locking (>0.6–0.7 W for the present design). Then, the second quarter-wave plate (after the beam splitter, birefringent plate and isolator; leftmost, Fig. 1a) should be rotated back and forth to sample a wide range of orientations. If mode-locking is not apparent, the half-wave plate should then be stepped a few degrees and rapid rotation of the second quarter-waveplate should be repeated. This process is iterated until a stable mode lock is observed. Note that cavity Q-switching often appears before and near the wave plate orientations suitable for mode-locking. Once the characteristic “cat-ear” spectrum appears, fine adjustments of the second quarter-wave plate can be used to adjust the spectrum and to suppress continuous wave background visible as a third peak centered between the steep sides of the spectrum. The angle of the birefringent filter may also be adjusted to tune the center wavelength of the pulse spectrum over a range of approximately 10 nm.

### Laser scanning microscopy

Linear excitation (single-photon) confocal and multiphoton excitation images were acquired using an Olympus FLUOVIEW FV3000 laser scanning microscope with a 30× (1.05 NA) or 60× (1.3 NA) silicone oil immersion objective. The dechirped output of the custom fiber laser was guided into the beam combining and scanning optics of the microscope using a periscope mirror assembly. De-scanned detectors were used for both linear and nonlinear imaging, with the pinhole opened to the maximum setting during multiphoton excitation using the custom fiber laser. The mode-locked pulse durations of the laser output were ~100–120 fs without pre-compensation to account for pulse dispersion and temporal broadening by the microscope optics. Assuming a total dispersion of ~5,000 fs^2^ (typical for microscope optics, including the objective lens^39^) the ML pulse durations are ~167–170 fs (*e.g*., see Equation 4 in Chapter 28 of ref.^40^). Linear imaging of the multicolor fluorescent microspheres (3 μm; FPMAS-30M9, Spherotech) was carried out using 405-, 488-, 514-, 561-, and 594-nm diode lasers. Hyperspectral image stacks of the microspheres were collected for linear excitation (514 nm) and using the custom fiber laser (1070 nm) for multiphoton excitation over a range of 525–745 nm in 10 nm steps. The second harmonic generation (445–545 nm) and rhodamine B (550–650 nm) image of the freshly excised chicken tissue specimen (Fig. 2d) is the maximum intensity projection of a 3D z-stack image series acquired by stepping the stage in 2-μm increments over a depth range of 30 μm. Analysis of the brine shrimp autofluorescence images using different modes of custom fiber laser operation were performed in ImageJ.

### Statistical analyses

Specific statistical tests are indicated in the figure captions and were carried out using GraphPad Prism (GraphPad Software). All reported *P* values are two-tailed. Parametric tests (one-way ANOVA with Tukey’s post hoc test) were used.

## Supplementary information

Supplementary Information

Supplementary Video 1

Supplementary Video 2

Supplementary Video 3

## Data Availability

The datasets generated during and/or analyzed during the current study are available from the corresponding author on reasonable request.

## References

[1] Zipfel WR, Williams RM, Webb WW (2003). Nonlinear magic: multiphoton microscopy in the biosciences. Nature Biotechnology.

[2] Helmchen F, Denk W (2005). Deep tissue two-photon microscopy. Nat Methods.

[3] Savage N (2010). Optical parametric oscillators. Nature Photonics.

[4] Xu C, Wise F (2013). Recent advances in fibre lasers for nonlinear microscopy. Nature Photonics.

[5] Kieu K, Wise F (2008). All-fiber normal-dispersion femtosecond laser. Optics express.

[6] Fekete J, Cserteg A, Szipőocs R (2009). All‐fiber, all‐normal dispersion ytterbium ring oscillator. Laser Physics Letters.

[7] Krolopp Á (2016). Handheld nonlinear microscope system comprising a 2 MHz repetition rate, mode-locked Yb-fiber laser for *in vivo* biomedical imaging. Biomedical Optics Express.

[8] Fermann ME, Hartl I (2013). Ultrafast fibre lasers. Nature Photonics.

[9] Szczepanek J, Kardaś TM, Michalska M, Radzewicz C, Stepanenko Y (2015). Simple all-PM-fiber laser mode-locked with a nonlinear loop mirror. Optics Letters.

[10] Bowen P, Singh H, Runge A, Provo R, Broderick NG (2016). Mode-locked femtosecond all-normal all-PM Yb-doped fiber laser at 1060 nm. Optics Communications.

[11] Freudiger CW (2014). Stimulated Raman scattering microscopy with a robust fibre laser source. Nature Photonics.

[12] Orringer DA (2017). Rapid intraoperative histology of unprocessed surgical specimens via fibre-laser-based stimulated Raman scattering microscopy. Nature Biomedical Engineering.

[13] Kieu K, Renninger W, Chong A, Wise F (2009). Sub-100 fs pulses at watt-level powers from a dissipative-soliton fiber laser. Optics letters.

[14] Prevedel R (2016). Fast volumetric calcium imaging across multiple cortical layers using sculpted light. Nature Methods.

[15] Tang, S., Liu, J., Krasieva, T. B., Chen, Z. & Tromberg, B. J. Developing compact multiphoton systems using femtosecond fiber lasers. *Journal of Biomedical Optics* **14**, 030508–030508–3 (2009). 10.1117/1.3153842 PMC2864591 19566289

[16] Li X (2016). High-power ultrafast Yb:fiber laser frequency combs using commercially available components and basic fiber tools. Review of Scientific Instruments.

[17] Perillo EP (2016). Deep *in vivo* two-photon microscopy with a low cost custom built mode-locked 1060 nm fiber laser. Biomedical Optics Express.

[18] Kong C (2017). Compact fs ytterbium fiber laser at 1010 nm for biomedical applications. Biomedical Optics Express.

[19] Runge AF, Aguergaray C, Broderick NG, Erkintalo M (2013). Coherence and shot-to-shot spectral fluctuations in noise-like ultrafast fiber lasers. Optics Letters.

[20] Churkin D (2015). Stochasticity, periodicity and localized light structures in partially mode-locked fibre lasers. Nature Communications.

[21] Chong A, Buckley J, Renninger W, Wise F (2006). All-normal-dispersion femtosecond fiber laser. Optics Express.

[22] Wise FW (2012). Femtosecond Fiber Lasers Based on Dissipative Processes for Nonlinear Microscopy. IEEE Journal of Selected Topics in Quantum Electronics.

[23] Xu C, Webb WW (1996). Measurement of two-photon excitation cross sections of molecular fluorophores with data from 690 to 1050 nm. J. Opt. Soc. Am. B.

[24] Kobat D (2009). Deep tissue multiphoton microscopy using longer wavelength excitation. Optics Express.

[25] Gao L, Zhu T, Wabnitz S, Liu M, Huang W (2016). Coherence loss of partially mode-locked fibre laser. Scientific Reports.

[26] Bale B, Kieu K, Kutz J, Wise F (2009). Transition dynamics for multi-pulsing in mode-locked lasers. Optics Express.

[27] Renninger W, Chong A, Wise FW (2010). Area theorem and energy quantization for dissipative optical solitons. J Opt Soc Am B.

[28] Ji N, Magee JC, Betzig E (2008). High-speed, low-photodamage nonlinear imaging using passive pulse splitters. Nature Methods.

[29] Hänsel W (2017). All polarization-maintaining fiber laser architecture for robust femtosecond pulse generation. Applied Physics B.

[30] Liu Z, Ziegler ZM, Wright LG, Wise FW (2017). Megawatt peak power from a Mamyshev oscillator. Optica.

[31] Sidorenko P, Fu W, Wright LG, Olivier M, Wise FW (2018). Self-seeded, multi-megawatt, Mamyshev oscillator. Optics Letters.

[32] Wang K (2012). Three-color femtosecond source for simultaneous excitation of three fluorescent proteins in two-photon fluorescence microscopy. Biomedical Optics Express.

[33] Horton NG (2013). *In vivo* three-photon microscopy of subcortical structures within an intact mouse brain. Nature Photonics.

[34] Wang K, Horton NG, Charan K, Xu C (2014). Advanced Fiber Soliton Sources for Nonlinear Deep Tissue Imaging in Biophotonics. IEEE Journal of Selected Topics in Quantum Electronics.

[35] Wright LG (2017). Multimode Nonlinear Fiber Optics: Massively Parallel Numerical Solver, Tutorial, and Outlook. IEEE Journal of Selected Topics in Quantum Electronics.

[36] Renninger W, Chong A, Wise F (2008). Dissipative solitons in normal-dispersion fiber lasers. Physical Review A.

[37] Wright LG, Christodoulides DN, Wise FW (2015). Controllable spatiotemporal nonlinear effects in multimode fibres. Nature Photonics.

[38] Agrawal, G. P. Pulse propagation in fiber, Third Edition. *Nonlinear Fiber Optics*. Chapter 2, 51–54 (Academic Press, 2001).

[39] Wolleschensky R, Feurer T, Sauerbrey R, Simon U (1998). Characterization and optimization of a laser-scanning microscope in the femtosecond regime. Applied Physics B.

[40] Denk, W., Piston, D. W. & Webb, W. W. Multi-photon molecular excitation in laser-scanning microscopy, Third Edition. [Pawley, J. B.] *Handbook of Biological Confocal Microscopy*. Chapter 28, 535–549 (Springer, 2006).

