# Supplementary material for: Custom fabrication and mode-locked operation of a femtosecond fiber laser for multiphoton microscopy
Source: Sci Rep. 2019 Mar 12;9:4233. doi: 10.1038/s41598-019-40871-5 (PMC6414530; doi:10.1038/s41598-019-40871-5)
Supplement: Supplementary file 1 — Supplementary Information [file 41598_2019_40871_MOESM1_ESM.pdf]

Supplementary Information

Custom fabrication and mode-locked operation of a femtosecond fiber laser for multiphoton microscopy

Nima Davoudzadeh, Guillaume Ducourthial, Bryan Q. Spring*

*email: b.spring@northeastern.edu

**Supplementary Figure 1**	Custom fs fiber laser design.
**Supplementary Figure 2**	Fiber fusion splicing.
**Supplementary Figure 3**	Diagram of the 31 MHz oscillator circuit.
**Supplementary Figure 4**	Diagram of the 70 MHz oscillator circuit and basic pulse characteristics.
**Supplementary Figure 5**	Pulse spectrum stability.
**Supplementary Table 1**	Femtosecond fiber laser parts.
**Supplementary Table 2**	Fiber splicing and pulse characterization instrumentation.
**Supplementary Table 3**	Pulse characteristics for exemplary custom-built femtosecond fiber lasers.
**Supplementary Video Legends**	
**Supplementary Notes 1–6**	
**Supplementary References**	

Supplementary Figure 1. Custom fs fiber laser design.

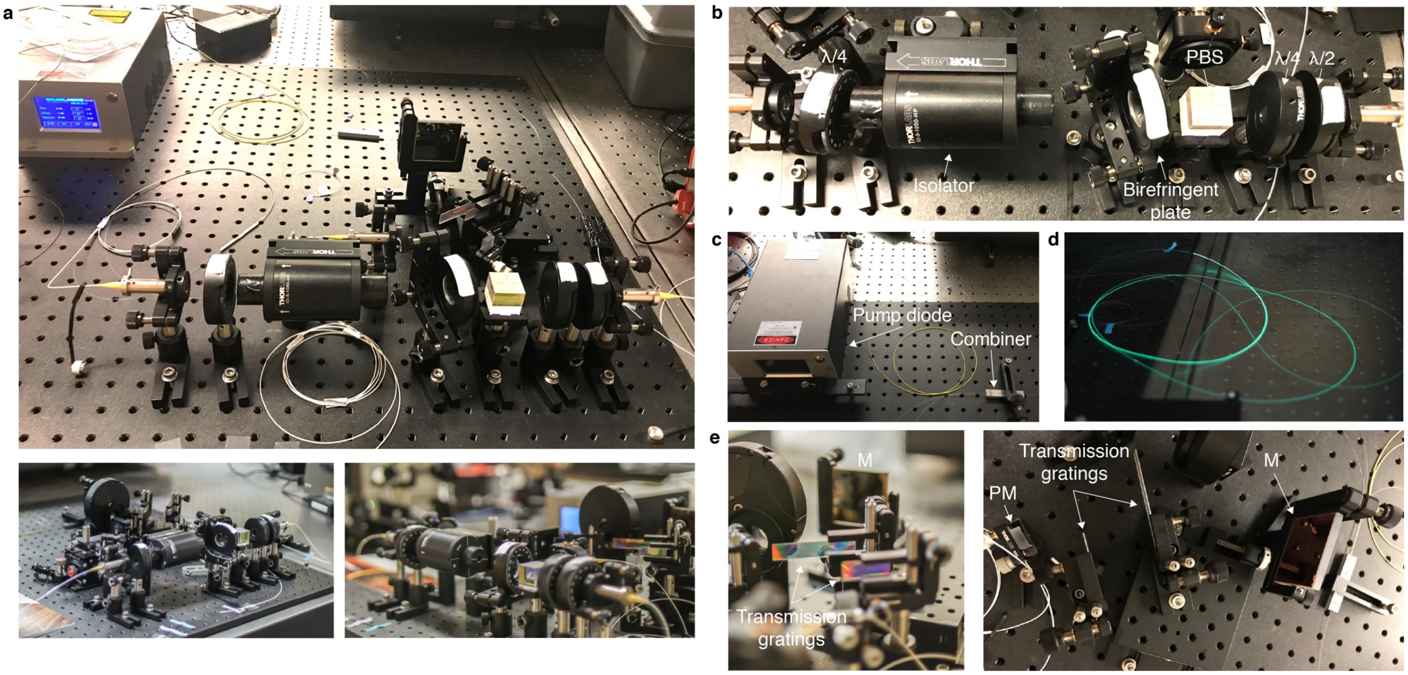

Supplementary Figure 1|(**a**) The entire custom fs fiber laser, including the pulse compression optics and pump diode, fits within a space of 26” × 16” (66 cm × 41 cm) with a maximum height of 7” (18 cm). Here, the pump diode (upper left corner) has been placed off to the side but could be placed adjacent to the free space optics. (**b**) Close up view of the free space components of the oscillator. λ/2, half-wave plate; λ/4, quarter-wave plate; PBS, polarizing beam splitter. (**c**) Close up of the pump diode and combiner positioned near the oscillator. (**d**) The Yb-doped gain fiber during laser operation. (**e**) A side- and overhead-view of the extra-cavity pulse compression optics. PM, pickoff mirror; M, mirror.

Supplementary Figure 2. Fiber fusion splicing.

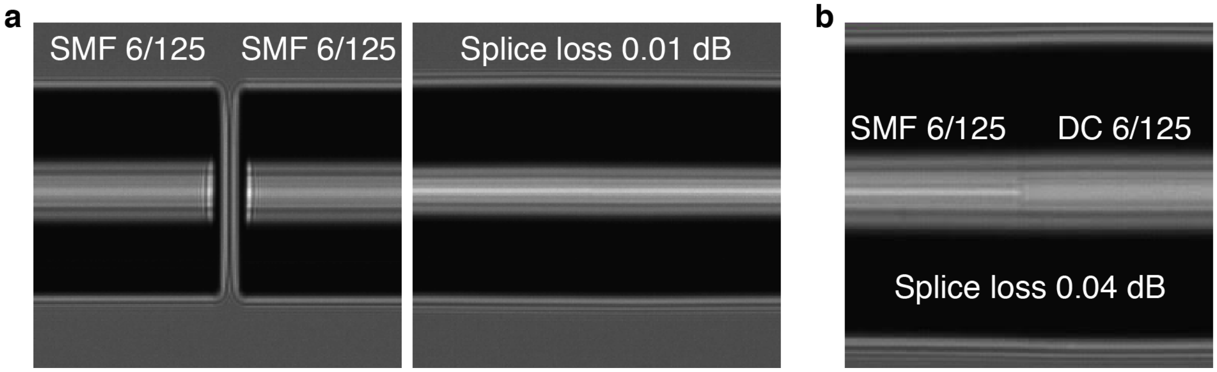

Supplementary Figure 2|(**a**) Pre- (*left*) and post-splice (*right*) views during fusion splicing of two identical single-mode fibers (SMF). The single mode core is surrounded by a dark cladding with a light line demarking the outer edge of the fiber. **(b)** An exemplary fusion splice of two dissimilar fibers (a SMF with a passive double clad, DC, fiber) used in the custom fs fiber laser oscillator. The splice losses are estimates determined in the fiber splicer software based on analysis of the core alignment.

Supplementary Figure 3. Diagram of the 31 MHz oscillator circuit.

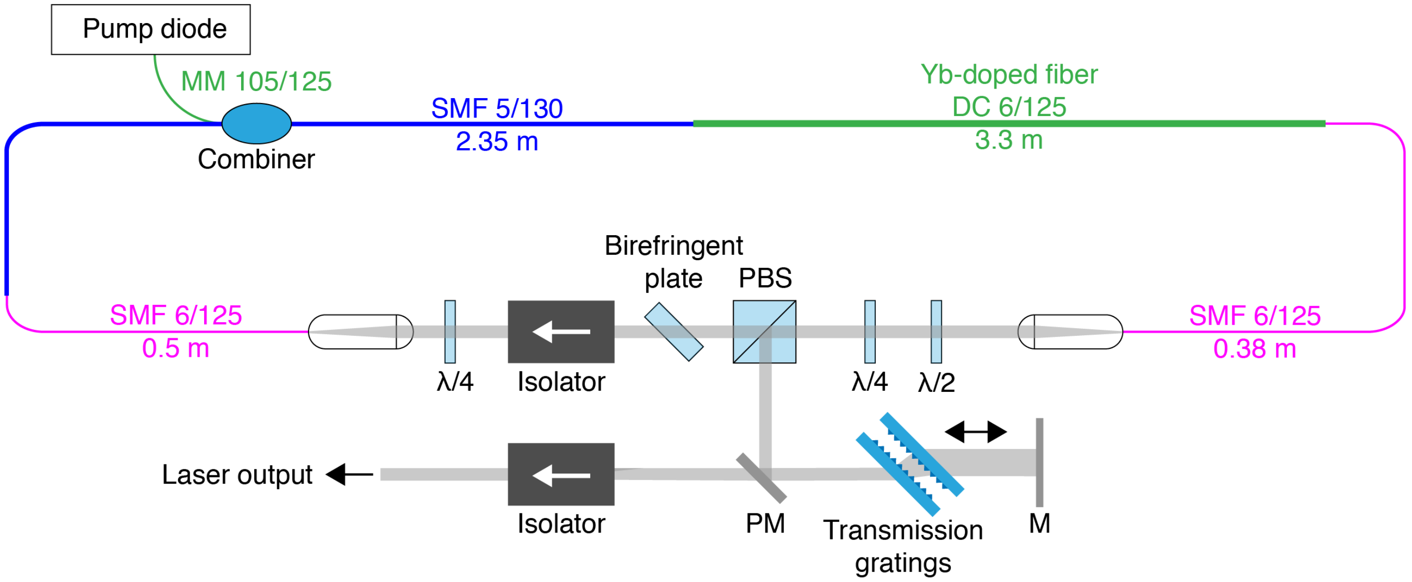

Supplementary Figure 3 | Detailed schematic of the 31 MHz laser oscillator. Each fiber type is represented by a distinct color in the diagram and labeled with the fiber type and length. The extra-cavity optics for pulse compression and oscillator isolation are repeated from Figure 1. λ/2, half-wave plate; λ/4, quarter-wave plate; PBS, polarizing beam splitter; PM, pickoff mirror; M, mirror.

Supplementary Figure 4. Diagram of the 70 MHz oscillator circuit and basic pulse characteristics.

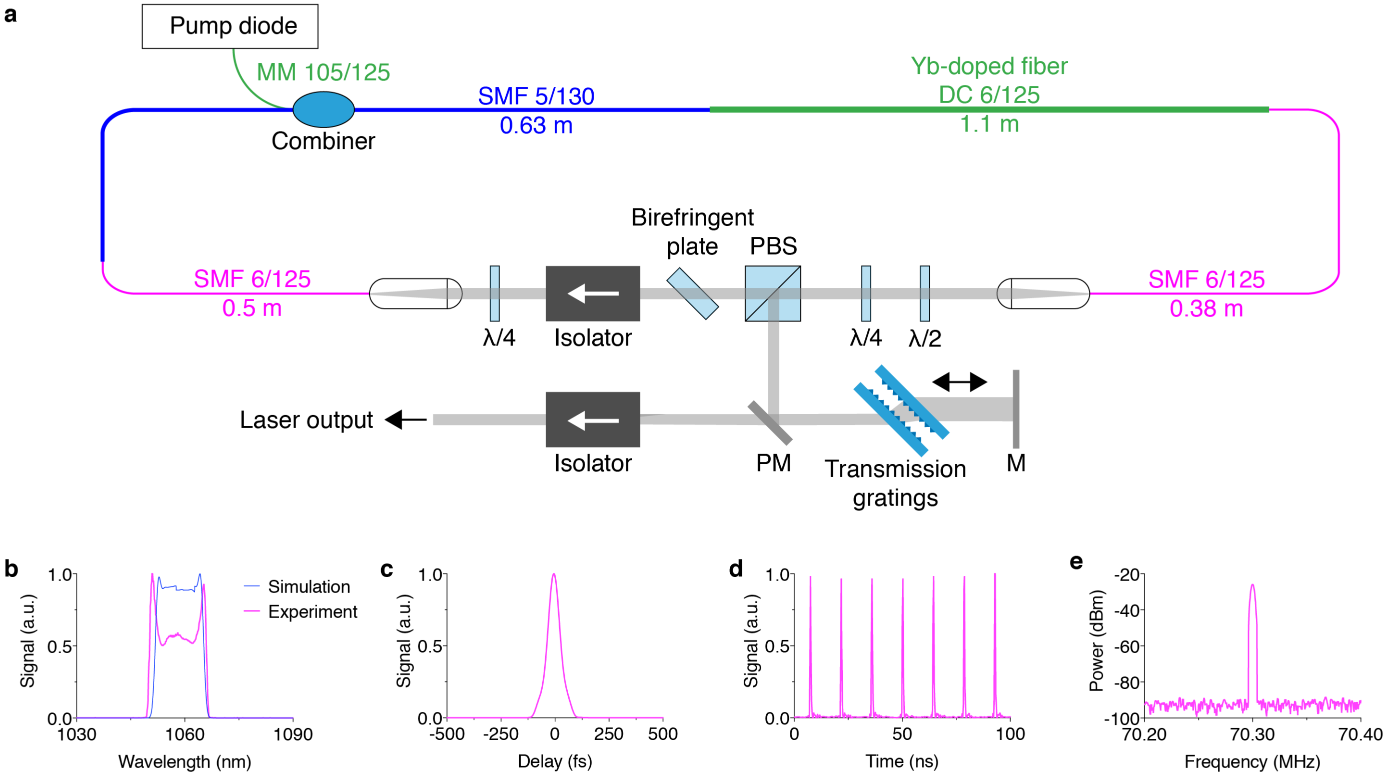

Supplementary Figure 4| (**a**) Detailed schematic of the 70 MHz laser oscillator. Each fiber type is represented by a distinct color in the diagram and labeled with the fiber type and length. The extra-cavity optics for pulse compression and oscillator isolation are identical to those of the 31 MHz oscillator (Fig. 1 and Supplementary Fig. 3). λ/2, half-wave plate; λ/4, quarter-wave plate; PBS, polarizing beam splitter; PM, pickoff mirror; M, mirror. (**b**) Simulated (*Methods*) and measured laser pulse spectra for the 70 MHz oscillator. (**c**) Intensity autocorrelation of a 73 fs laser pulse (full-width half-maximum) after dechirping the output of the 70 MHz oscillator. (**d**) Measured pulse train and (**e**) radio frequency power spectrum for the 70 MHz oscillator.

Supplementary Figure 5. Pulse spectrum stability.

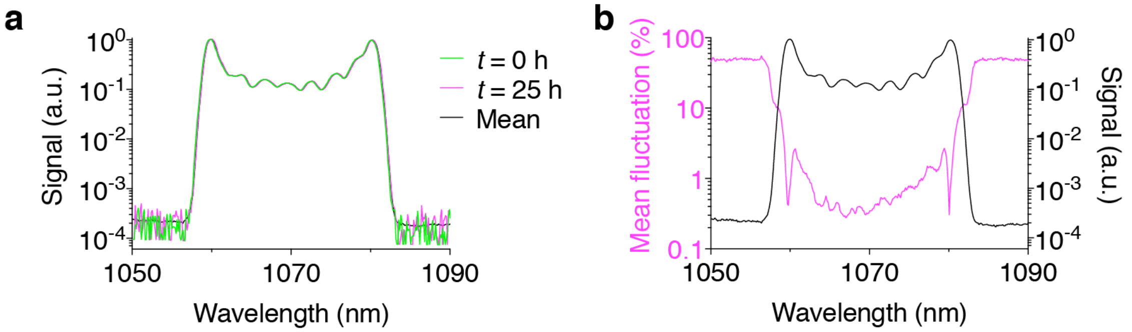

Supplementary Figure 5| The dechirped pulse spectrum of the custom fs fiber laser (31 MHz pulse repetition rate) was measured every 75 seconds over a period of approximately 25 h using an optical spectrum analyzer. (**a**) The initial pulse spectrum (t = 0 h), the pulse spectrum 25 h later (t = 25 h) and the mean pulse spectrum (over the entire 25 h period) are shown for comparison. Note the logarithmic scale of the signal axis. (**b**) Overlay of the mean intensity fluctuation size over the 25 h period at each wavelength (the standard deviation divided by the mean) with the mean pulse spectrum.

Supplementary Table 1 | Femtosecond fiber laser parts.

Description	Supplier	Part number	Quantity	Price[a]	Details
Pump diode					
High power multimode fiber pump module	Gauss Lasers (China)	Pump-MM-976-10	1	$1,800	976 nm, 10 W
High power pump and signal combiner	ITF Technology (Canada)	MMC02112DF1	1	$315	(2+1)×1, 5/130 μm signal and output fiber
Fiber					
Ytterbium-doped single mode double clad fiber	Thorlabs (USA)	YB1200-6/125DC	3 m	$202	7 μm mode field diameter (MFD; $67.30/m)
Fiber collimators	OZ Optics (Canada)	LPC-08-1064-6/125-S-1.6-7.5AS-60-X-1-2-HPC	2	$2,000	($1,000 each)
Passive double clad fiber (5/130 μm)	ITF Technology	MMC02112DF1	3 m	Included with above combiner	4.8 μm MFD
Single mode fiber (6/125 μm)	OZ Optics	LPC-08-1064-6/125-S-1.6-7.5AS-60-X-1-2-HPC	1 m	Included with above collimators	6.2 μm MFD
Intra-cavity optics & optomechanics					
Free-space isolator	Thorlabs	IO-5-1050-HP	1	$2,540	40 W max
Polarizing beamsplitter	Thorlabs	PBS253	1	$222	
Beamsplitter mount	Thorlabs	BSH1/M	1	$47	
Quartz birefringent filter plate	Newlight (Canada)	BIR1060	1	$279	6 mm thick, 10 nm bandwidth
Half waveplate	Union Optics (China)	WPZ2312	1	$75	
Quarter waveplates	Union Optics	WPZ4312	2	$150	($75 each)
Waveplate rotation mount	Thorlabs	RSP1/M	4	$344	Mounts for the waveplates and the birefringent filter ($86 each)
Extra-cavity pulse compression & optics					
Transmission diffraction grating 1	LightSmyth (USA)	T-1000-1040-3212-94	1	$395	31.8 mm × 12.3 mm
Transmission diffraction grating 2	LightSmyth	T-1000-1040-60×12.3-94	1	$795	60 mm × 12.3 mm
Free-space isolator	Thorlabs	IO-3D-1050-VLP	1	$1,565	0.7 W max
Standard optical components					
Index matching gel, adapters, mirrors, posts, mounts, and translational stage	Thorlabs	Part number (quantity): G608N3 (1), TR6-P5 (3), AD12NT (2), PFSQ20-03-M01 (1), PFSQ05-03-M01 (1), KMS (1), KM100C (1), KM100CL (1), KM200S (1), LT1 (1), LT101 (1), UPH2-P5 (1), UPH3-P5 (2)		$1,420	
Breadboard	Newport (USA)	SA2-30X30		$654	Solid aluminum plate, 30 in. x 30 in.

[a]Prices at the time of publication.

Supplementary Table 2 | Fiber splicing and pulse characterization instrumentation.

**Description**	**Supplier**	**Part number**	**Price**	**Details**
Fiber splicing				
Factory fusion splicer	AFL (USA)	FSM-100P	$34,675	new
Advanced optical fiber cleaver	AFL	CT-100	$3,330	new
Laser pulse characterization				
Autocorrelator	Femtochrome (USA)	FR-103XL/IR/FA/CDA	$10,190	new
Half waveplate	Union Optics	WPZ2312	$75	new
Waveplate rotation mount	Thorlabs	RSP1/M	$86	new
Fiber collimator	OZ Optics	LPC-08-1064-6/125-S-1.6-7.5AS-60-X-1-2-HPC	$1,000	new
Optical spectrum analyzer	Keysight	HP/Agilent 70951B	$5,700	refurbished
Fiber-coupled, high-speed photodiode detector	Thorlabs	DET08CFC	$350	InGaAs, 5 GHz, new
Oscilloscope	Keysight (USA)	Agilent 54845A	$2,950	1.5 GHz, used
RF spectrum analyzer	Tektronix (USA)	RSA306B	$3,382	new
Standard optical components				
Single mode fiber coupler	AFW (Australia)	FOSC-2-64-30-L-1-H64-2	$80	new
Adapter, beamsplitter, mirrors, posts, mounts, and fiber collimators	Thorlabs	Part number (quantity): KM100 (6), AD10NT (1), BSH1 (1), BS014 (1), CFS18-1064-FC (1), PF10-03-P01-10 (1 pack of 10)	$1,524	new

Supplementary Table 3 | Pulse characteristics for exemplary custom-built femtosecond fiber lasers.

**Fiber laser design**	**Mode-lock verified?**	**Fabrication protocol?**	**Parts list? (total cost)**	**Oscillator output power (pulse energy)**	**Repetition rate**	**Compressed pulse duration**	**Power stability quantified? (drift)**
This report	Yes	Yes	Yes ($13,000)	1 W (14 and 33 nJ)	30 and 70 MHz	70 fs	Yes (< ±3.5% over 24 h)
Kong et al. 2017 (ref. 18)	Yes	No	Yes ($6,000+)[a]	0.007 W (0.09 nJ)	80 MHz	294 fs	Yes (< ±2% over 24 h)
Perillo et al. 2016 (ref. 17)	No[b]	Yes	Yes ($13,000+)[a]	1 W (25 nJ)	40 MHz	81 fs	No
Li et al. 2016 (ref. 16)	Yes	Yes	Yes (N.R.)	0.04 W (0.4 nJ)	87 MHz	85 fs	Yes (N.R.)
Bowen et al. 2016 (ref. 10)	No	No	No	0.004 W (0.3 nJ)	11.6 MHz	360 fs	No
Szczepanek et al. 2015 (ref. 9)	Yes	No	No	0.05 W (3.5 nJ)	15 MHz	220 fs	Yes (< ±3% during vibration and heating)
Fekete et al. 2009 (ref. 6)	Yes	No	No	0.01 W (0.2 nJ)	43 MHz	195 fs	No
Kieu et al. 2009 (ref. 13)	Yes	No	No	2.2 W (31 nJ)	70 MHz	80 fs	No
Chong et al. 2007 (ref. 41)	Yes	No	No	0.3 W (26 nJ)	12.5 MHz	165 fs	No

[a]These parts lists are incomplete (*i.e.*, the parts lists are missing optomechanics, the extra-cavity isolator and other components).
[b]The published pulse characterization data suggest that the laser was not fully mode-locked. N.R. indicates not reported.

Supplementary Video Legends

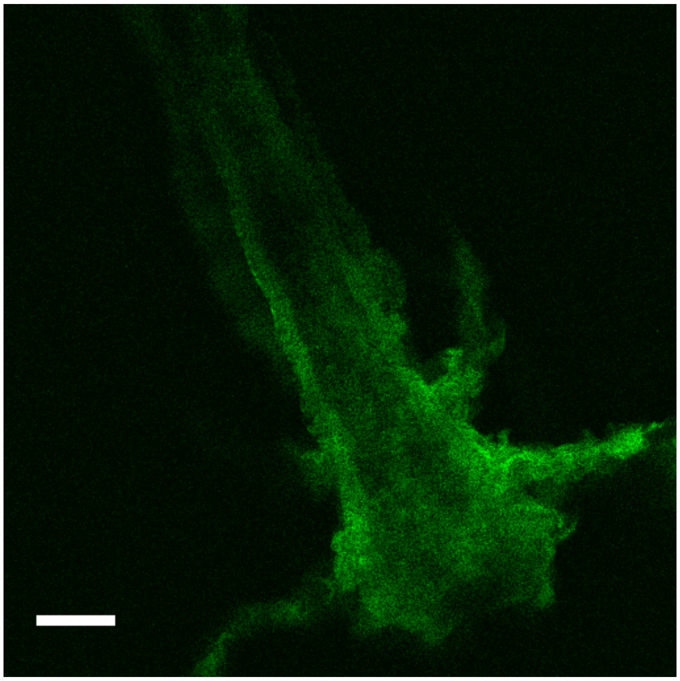

Supplementary Video 1. Mode-locked (ML) custom fiber laser operation. ML multiphoton microscopy time series of an unstained, autofluorescent brine shrimp specimen with uniform pulses. The images were acquired at 1 frame per second (1 fps), and the video playback is sped up 5× (5 fps). Scale bar, 50 μm.

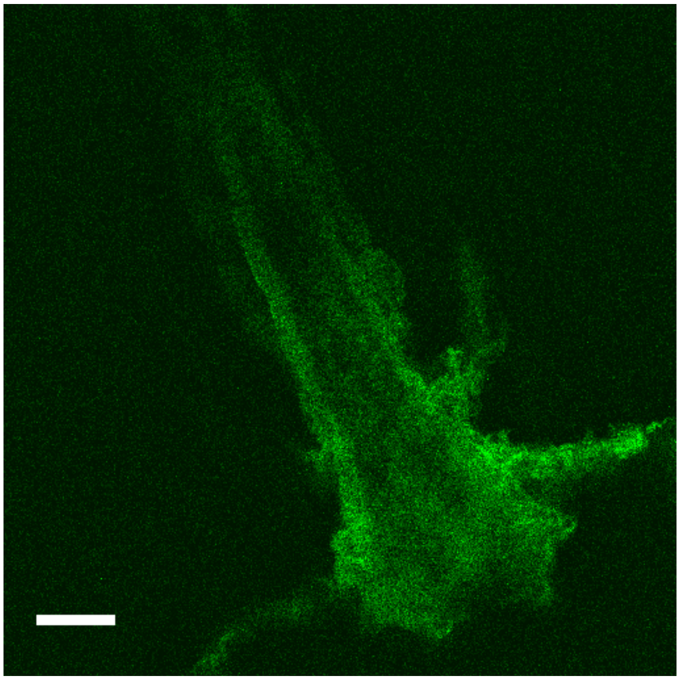

Supplementary Video 2. Partially mode-locked (PML), noise-like pulse custom fiber laser operation. PML multiphoton microscopy time series of an unstained, autofluorescent brine shrimp specimen indicating degraded signal-to-noise compared to ML laser operation. The images were acquired at 1 frame per second (1 fps), and the video playback is sped up 5× (5 fps). Scale bar, 50 μm.

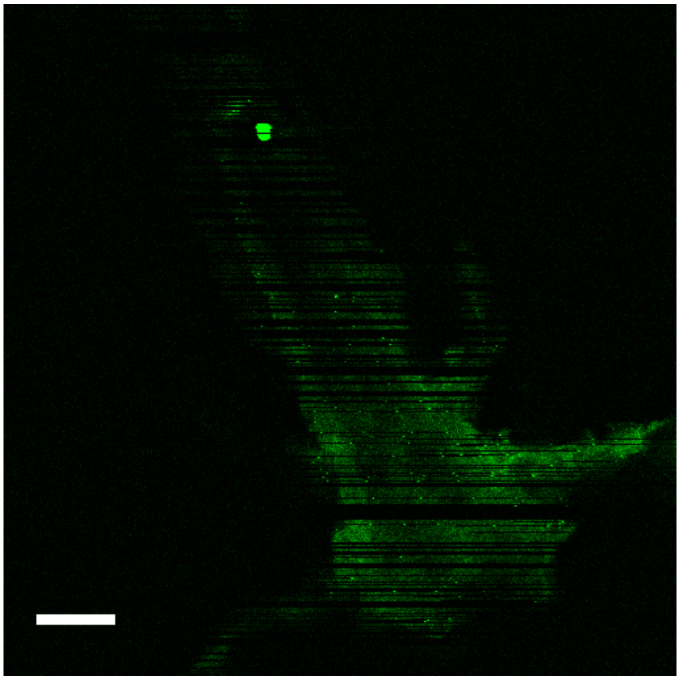

Supplementary Video 3. Non-mode-locked (NML), unstable Q-switching custom fiber laser operation. NML (Q-switching) multiphoton microscopy time series of an unstained, autofluorescent brine shrimp specimen with stochastic signal generation and photodamage during the laser scan due to fluctuations in pulse duration and amplitude. The images were acquired at 1 frame per second (1 fps), and the video playback is sped up 5× (5 fps). Scale bar, 50 μm.

Supplementary Notes

Supplementary Note 1. Custom-built Ti:sapphire oscillators can be low-cost and do not require investment in fiber splicing equipment. As an example, Squier and colleagues demonstrated that direct-diode pumping of a Ti:sappire femtosecond laser is possible using low-cost commercial blue diode lasers (<\$100), which can be applied to replace expensive green sources commonly sold as part of solid-state femtosecond laser systems (>\$25,000)[42].

Supplementary Note 2. Solid-state fs lasers are used in ophthalmology for blade-free, laser-assisted *in situ* keratomileusis (LASIK)[43]. Several commercial systems are in clinical use albeit with relaxed specifications (longer pulse durations and reduced repetition rates) than those required for multiphoton imaging (<150 fs and 10–100 MHz). For example, the iFS Advanced Femtosecond Laser (Johnson & Johnson; 600–800 fs and 150 kHz) and the VisuMax (Carl Zeiss Meditec; 220–580 fs and 500 kHz) are two products for LASIK approved by the US Food and Drug Administration.

Supplementary Note 3. The center wavelength of the laser (1060–1070 nm) is ideal for long-wavelength, deep multiphoton excitation of several fluorophores and fluorescent protein sensors commonly used in the life sciences. Examples of compatible fluorescent probes include Alexa Fluor 546 and 568[44] as well as yellow and red fluorescent protein variants (*e.g.*,YFP, DsRed and RCaMP)[17,45,46]. Green fluorescent protein (GFP) and its functional counterpart for calcium sensing (GCaMP) cannot be used directly as the peak two-photon excitation wavelength is ~920 nm[44,45]. However, YFP and RCaMP can be substituted for GFP and GCaMP, respectfully.

Supplementary Note 4. The Wise Research Group (Cornell University) led development of ANDi dissipative soliton fiber lasers, and the group webpage (http://wise.research.engineering.cornell.edu/dissipative-soliton/, 2018) provides an accessibly entry point with animations of the pulse shaping physics as a pulse propagates through the various components of the oscillator.

Supplementary Note 5. Multi-pulsing occurs in ANDi fiber lasers when the pulse energy is increased due to energy quantization and an upper limit to the pulse energy of dissipative solitons[27]. The single pulse then undergoes bifurcation in favor of a multi-pulse solution that divides the pulse energy. In the present fs fiber laser design, multi-pulsing ML operation may be observed at pump powers as low as 1 W, however, it is straightforward to tune the laser to single-pulse ML operation up to pump powers of ~3.6 W. A potential nuance with multi-pulsing is that there exists a small range of pump powers at which a chaotic transition between stable single and multi-pulsing exists (for each transition from N to N+1 pulses) that could compromise image quality. However, the chaotic transition is generally hard to capture experimentally without significant effort to achieve refined control of the pump power[26].

Supplementary Note 6. An excellent video tutorial that describes the process for finding mode-locked operation of an ANDi femstosecond fiber laser is available online (https://www.youtube.com/watch?v=Zd5OH2iyno0; Logan Wright, Wise Research Group, 2018).

Supplementary References

41. Chong, A., Renninger, W. & Wise, F. All-normal-dispersion femtosecond fiber laser with pulse energy above 20nJ. *Optics Letters* **32,** 2408–2410 (2007).

42. Young, M. D., Backus, S., Durfee, C. & Squier, J. Multiphoton imaging with a direct-diode pumped femtosecond Ti:sapphire laser. *Journal of Microscopy* **249,** 83–86 (2013).

43. Callou, T. *et al.* Advances in femtosecond laser technology. *Clinical Ophthalmology* **10,** 697–703 (2016).

44. Mütze, J. *et al.* Excitation Spectra and Brightness Optimization of Two-Photon Excited Probes. *Biophysical Journal* **102,** 934–944 (2012).

45. Drobizhev, M., Makarov, N. S., Tillo, S. E., Hughes, T. E. & Rebane, A. Two-photon absorption properties of fluorescent proteins. *Nature Methods* **8,** 393–399 (2011).

46. Akerboom, J., Calderón, C. N., Lin, T. L. & Wabnig, S. Genetically encoded calcium indicators for multi-color neural activity imaging and combination with optogenetics. *Frontiers in Molecular Neuroscience* **6,** 1–29 (2013).
